# Surface Treatment of Industrial-Grade Magnetite Particles for Enhanced Thermal Stability and Mitigating Paint Contaminants

**DOI:** 10.3390/nano11092299

**Published:** 2021-09-04

**Authors:** Mohua Sinhababu, Anurag Roy, Narendra Kumar, Monojit Dutta, Senthilarasu Sundaram, Smagul Karazhanov, Gopalkrishnan Udayabhanu

**Affiliations:** 1 Department of Chemistry, Indian Institute of Technology (Indian School of Mines), Dhanbad 826004, India; 2 Research & Development, Tata Pigments Limited, Boulevard, Sakchi, Jamshedpur 831002, India; narendra@tatapigments.co.in; 3 Environment and Sustainability Institute, University of Exeter, Penryn Campus, Cornwall TR10 9FE, UK; a.roy30@exeter.ac.uk (A.R.); s.sundaram@exeter.ac.uk (S.S.); 4 Research & Development, Tata Steel Limited, Jamshedpur 831007, India; monojitdutta@tatasteel.com; 5 Institute for Energy Technology (IFE), P.O. Box 40, 2027 Kjeller, Norway

**Keywords:** magnetite, thermal stability, environmental contamination, SHMP-treated, XRD, calcined, color difference value

## Abstract

Pigments can retain their color for many centuries and can withstand the effects of light and weather. The paint industry suffers from issues like aggressive moisture, corrosion, and further environmental contamination of the pigment materials. Low-cost, long-lasting, and large-scale pigments are highly desirable to protect against the challenges of contamination that exist in the paint industry. This exploratory study reinforces the color and thermal stability of industrial-grade (IG) magnetite (Fe_3_O_4_). IG Fe_3_O_4_ pigments were further considered for surface treatment with sodium hexametaphosphate (SHMP). This metaphosphate hexamer sequestrant provides good dispersion ability and a high surface energy giving thermal and dust protection to the pigment. Various physicochemical characterizations were employed to understand the effectiveness of this treatment across various temperatures (180–300 °C). The X-ray diffraction, Raman, and X-ray photoelectron spectroscopy techniques signify that the SHMP-treated Fe_3_O_4_ acquired magnetite phase stability up to 300 °C. In addition, the delta-E color difference method was also adopted to measure the effective pigment properties, where the delta-E value significantly decreased from 8.77 to 0.84 once treated with SHMP at 300 °C. The distinct color retention at 300 °C and the improved dispersion properties of surface-treated Fe_3_O_4_ positions this pigment as a robust candidate for high-temperature paint and coating applications. This study further encompasses an effort to design low-cost, large-scale, and thermally stable pigments that can protect against UV-rays, dust, corrosion, and other color contaminants that are endured by building paints.

## 1. Introduction

Magnetite (Fe_3_O_4_), or black iron oxide, is a standard ferrite with a cubic inverse spinel structure [1]. It has unique magnetic properties due to the transfer of electrons between Fe^2+^ and Fe^3+^ ions in the octahedral sites. Among the three most essential iron oxides used as pigments (red, yellow, and black), red iron oxide Fe_2_O_3_ (hematite) demonstrates the highest thermodynamic stability up to 1200 °C, even at elevated temperatures [2]. In the temperature range typically encountered in paint and coating applications, its color remains practically unchanged. In contrast, yellow iron oxide FeO(OH) (goethite) and black iron oxide Fe_3_O_4_ (magnetite) pigments are stable up to 180 °C, and eventually convert to red α-Fe_2_O_3_ (hematite) at elevated temperatures [3].

Magnetite (Fe_3_O_4_) offers good color strength, opacity, weather and chemical resistance, and durability to substrates. In addition, they provide stable coloring effects for end-user applications employed in numerous industries worldwide. Magnetite (Fe_3_O_4_) has been widely utilized in pigments, electrophotographic development, catalysts [4], high-density magnetic recording media [5], microwave devices [6], ferrofluids in heat transfer [7], and biomedical applications mainly for cancer treatment [8,9,10]. Multifunctional Fe_3_O_4_@TiO_2_@Ag composites have been investigated for use in the early diagnosis of cancer [11,12]. As a black oxide pigment, magnetite has been used as a coloring agent in paints for cooking furnaces, chimneys, ovens, stoves, floor coatings, steam generation equipment such as boilers, powder coatings, coil coatings [13], heat shrink applications, induced draught (ID) fans, and radiators. They have also been found in applications in plastic masterbatches, ceramics, leatherette, wood coatings, paper, and in the textile industry as an inorganic and eco-friendly substitute. The curing temperature of the paint used for these end applications is in the range of 180–300 °C. Upon calcination above 180 °C, magnetite starts oxidizing and transforming to maghemite (γ-Fe_2_O_3_), corresponding to the oxidation of ferrous ions without changing the spinel structure. On further calcination, maghemite is transformed to hematite (α-Fe_2_O_3_) with a lattice rearrangement, representing the spinel structure’s stability limit [3]. Cuenca et al. (2016) reported that Fe_3_O_4_ powders calcined above 200 °C were transitioning to γ-Fe_2_O_3_. Oxidation of Fe_3_O_4_ at temperatures around 200–300 °C induces the phase change to γ-Fe_2_O_3_ [14]. The thermal stability property of magnetite up to 300 °C needs to be reinforced for its use in high-temperature applications, which would play a key differentiator.

Remarkable compound annual growth (CAGR) in the paint industry and the recovering economies of developed countries are critical factors contributing to the growth in iron oxide pigment consumption worldwide. The global paint and coating industry is expected to be valued at USD 179 billion by 2025. There is an increasing demand for paints and coatings in the construction, automotive, general industrial, coil, and packaging industries [15]. Therefore, an intense focus on research and development activity is essential in this sector.

As Wei et al. (2008) reported, several experiments have been conducted to functionalize the magnetite surface using synthetic polymer, and organic and inorganic materials [16]. Several researchers prepared thermal stable black pigment (cobalt chromite black—Pigment Black 27, and copper chromite black—Pigment Black 28 [17]) using chromium, which is very expensive and hazardous to the environment [18]. Moreover, manganese ferrite black (Pigment Black 26) has been used in applications requiring higher temperature stability [17], but its preparation requires high temperatures and annealing [19]. Xing et al. (2021) reported the usage of Fe_5_C_2_ nanoparticles as a hyperthermia heat probe, with dual magneto-photo-thermal therapeutic features [20]. A thin coating of carbon on the surface of Fe can effectively enhance the magnetothermal heating if the applied alternating magnetic field amplitude is equivalent to the coercivity values.

Therefore, our aim is to develop thermally stable pigments employing a cost-effective and environmentally friendly process. An innovative approach to functionalize the Fe_3_O_4_ surface to improve its thermal stability and its potential usage in high-temperature coating applications needs to be designed. Phosphate molecules have a strong affinity for transition metal oxide surfaces, especially iron oxides [21,22,23]. Recently, sodium hexametaphosphate (SHMP), a mixture of sodium metaphosphate salts used as a food additive, has been widely used as a stabilizer for generating various nanoparticles, such as Au, BaSO_4_, ZnCdS, and ZnS: Cu^2+^ [24,25,26,27]. Mehrdad et al. employed SHMP to provide heat stability to whey protein-based drinks [28]. SHMP is used as a deflocculant and dispersing agent to break down clay. It also has anti-staining and tartar prevention properties.

Recent studies have shown that manufacturing of paint and coating can produce hazardous air pollutants, including toxic air pollutants and volatile organic compounds (VOC) [29]. Unfortunately, this causes ground-level ozone (smog), which is greatly responsible for respiratory problems. Paint and Coating manufacturing facilities emit pollutants such as hazardous air pollutants (HAPs), volatile organic compounds (VOC), and particle pollution (dust).In England recently, a coat of paint on the walls of a house was observed to help reduce CO_2_ emissions and improve air quality by reducing chemicals, pollutants, and harmful pathogens [30]. On one of Manila’s busiest roads, Pacific Paint discovered that one square meter of wall painted with KNO_x_OUT could remove up to 160 g of NO_x_ per year, which is comparable to the air-cleaning impact of a mature tree [31]. The black textile dyes that are released from industries compromise the aesthetic quality of water bodies, increase biochemical and chemical oxygen demand (BOD and COD), impair photosynthesis, inhibit plant growth, enter the food chain, provide recalcitrance and bioaccumulation, and may promote toxicity, mutagenicity, and carcinogenicity [32]. Black color dye, primarily used in the textile and leather industries as a coloring agent, causes the soil to become toxic and can bring about allergies such as contact dermatitis and respiratory diseases, allergic eye reactions, skin irritations, and irritation to the mucous membrane of the upper respiratory tract. These diseases are commonly prevalent in the workers who are dyeing the clothes, as they have maximum exposure to it all day. Carbon black, which is commonly used as a black pigment, has been classified as carcinogenic by the International Agency for Research on Cancer. Inhalation of carbon black is associated with respiratory and cardiovascular disease, cancer, and even congenital disabilities. In addition, the waste materials from various petrochemical industries have found a use in manufacturing of decorative paints for future reuse in a sustainable manner [33]. Therefore, a non-hazardous, thermally stable color, water protective, long-lasting, and economical pigment or coating material could mitigate these problems.

In the present study, we are reporting the surface modification of an industrial-grade (IG) Fe_3_O_4_ pigment employing SHMP, focusing on improving its thermal stability and colorant property. Bare and SHMP-treated IG Fe_3_O_4_ were calcined at different temperatures ranging from 180–300 °C. Various characterizations were performed based on the structure, surface morphology, and thermal and color stability behavior of the bare IG Fe_3_O_4_ and SHMP-treated Fe_3_O_4_ to understand the effectiveness of the treatment. The surface-treated Fe_3_O_4_ offers potential stability against paint corrosion degradation and mitigates air pollutant contamination in a better and more sustainable manner. This study could further develop an innovative approach to tumor cell studies for cancer treatment [9,10,34,35,36,37] in the future.

## 2. Materials and Methods

### 2.1. Materials

**Figure 1:**
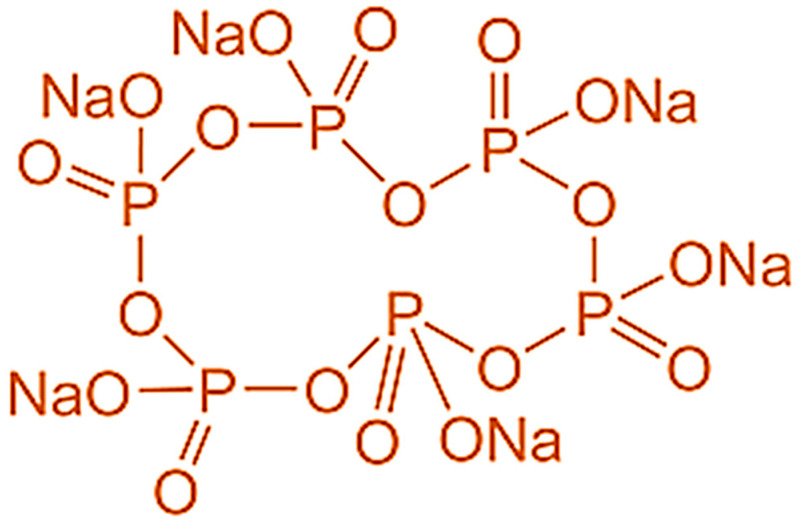
Chemical structure of sodium hexametaphosphate (SHMP).

Industrial-grade (IG) synthesized black iron oxide pigment, Fe_3_O_4_, was collected from Tata Pigments Limited (Jamshedpur, India). All the employed resins, solvents, and additives were fine chemical grade and were products of various commercial companies. Sodium hexametaphosphate ((NaPO_3_)_6_), SHMP (68%), was procured from Loba Chemie and was of laboratory reagent grade. The chemical structure of the SHMP is shown in Figure 1.

### 2.2. SHMP Treatment of Industrial-Grade Fe_3_O_4_

An amount of 20% *w*/*v* solution (SHMP and deionized water) was prepared and stirred with a magnetic stirrer for 15 min to form a clear transparent solution. Next, 100 g of dry IG Fe_3_O_4_ powder was mixed with 500 mL of deionized water for 10 min to form a homogenous slurry. The aqueous solution of Fe_3_O_4_ was prepared in three separate beakers of 1 L each for treatment with SHMP at different ratios; that is, 85:15, 88:12, and 90:10 of Fe_3_O_4_: SHMP *w*/*w* solution. The solution of Fe_3_O_4_ and SHMP (at different *w*/*w* ratios) was stirred for a further 30 min to obtain a consistent slurry. The slurries collected from different beakers were filtered separately using grade 2 filter paper through a Buchner funnel, which is associated with a rotary vacuum pump. The wet cake residues of the treated pigment material were collected in petri dishes.

### 2.3. Material Characterization

Scanning electron microscope (SEM) measurements were performed on Quanta FEG 650 microscopes from UK with an energy-dispersive X-ray detector (Bruker model no. X flash 6160 from Germany). A copper metal mold was taken, and carbon tape was labelled over the copper mold’s surface, which was conductive. Bare Fe_3_O_4_ and treated Fe_3_O_4_ powders were sprinkled over the carbon tape, and the loose particles were removed using a hot air drier. The sample was then placed in a baking unit to remove the moisture for approximately 30 min. Next, it was extracted from the baking unit and immediately placed in a desiccator for cooling. The top surface was further made conductive by coating it with a mixture of gold and palladium of a thickness less than 100 nm. The voltage was kept at 5–10 kV, having a resolution of 1.5 nm. The magnifications used for the imaging were 25,000X and 100,000X, respectively, employing an Everhart–Thornley detector (ETD), as the image was taken from the top surface.

A low vacuum atmosphere was created to conduct the energy-dispersive X-ray spectroscopy (EDX) analysis, as the samples that were taken for analysis were powder samples of Fe_3_O_4_. First, the beam was switched on. The chamber pressure was kept at around 50–100 Pa, a high voltage of 15 KV was applied with a magnification of 25,000X, and the working distance was kept as close to minimum as possible for a better analysis.

Bare IG Fe_3_O_4_ and the treated Fe_3_O_4_ were prepared as pressed powders and mounted in a stainless-steel sample holder. The powder X-ray diffraction (XRD) patterns were recorded on a Philips PANalytical X’Pert PRO diffractometer from Netherlands, utilizing Cu Kα radiations, operating at 45 kV and 40 mA. XRD diffraction patterns were analyzed in the range of 10–90° at a scan speed of 0.00334° s^−1^ with a 0.5° divergence slit size. Phase identification was carried out by comparison with the Inorganic Crystal Structure Database (ICSD).

Raman Spectroscopy was performed by a Renishaw inVia (M-9836-3991-04-A) optical microscope (Leica model DM2700) from UK with a 785 nm solid-state diode laser with 0.1% power and an exposure time of 60 s. However, due to the nature of the measurement, phase change can be induced, from magnetite to hematite, caused by excess heat from the laser, thus great care needs to be taken to measure these materials. We took reasonable care to avoid this by selecting the lowest laser power possible with the instrument, which was 0.1%.

A zeta potential analyzer (Malvern Panalytical, Zetasizer v7.11, Malvern, UK) measured the zeta potential of bare Fe_3_O_4_ and treated Fe_3_O_4_ particles at three different concentrations at 25 °C. Before measurement, the powder sample suspension was diluted to a concentration of approximately 0.009 mg Fe. The pH of the suspension was adjusted to the desired values by employing 0.1 mol L^−1^ of HCl NaOH. To estimate the suspension’s stability at different pH levels, the optical absorbencies of the suspension at 420 nm were recorded on a Hitachi U-4100 spectrophotometer. The particle size distribution was analyzed through an analyzer (Malvern Panalytical, Mastersizer v3.63, UK) that measured the particles in a range from nanometers to millimeters. Distilled water was taken as the dispersant with a refractive index of 1.33 and a scattering model, Mie laser, obscuration of 41%.

XPS measurement was carried out on an ESCALAB 250 Xi photoelectron spectrometer (Thermo Fisher Scientific from Lenexa, KS, USA) equipped with a monochromatic Al Kα source at 1486.6 eV under ultra-high vacuum (1.0 × 10^−9^ Pa). The energy resolution of the scan was 0.05 eV. The diameter of the sample spot size was ~650 μm. The charge effects of the spectra were corrected by using the C 1s peak at 284.8 eV.

The delta-E color difference (dE) was recorded in a Premier Colorscan colorlab+ color-matching software licensed to Tata Pigments Ltd. (Model-SS 5100A). The samples were measured in reflectance mode. Color (visible light: 300–700 nm) was measured between maximum reflectance (100% white) and maximum absorption (100% black). The spectrophotometer needs to be calibrated with a white tile every time it is turned on. In quality control, the batch sample was compared with the standard sample taken for reference, and the subsequent output was recorded as dE in color tone. A detailed analysis of the samples was explored regarding their hue, lightness, saturation, and overall position in the Color Space Index. The experiments for dE measurements were repeated three times to gain reliability.

Thermogravimetry (TG) was measured with a thermo-gravimetric analyzer (TGA, Q500, Hertfordshire, UK) at a heating rate of 5 °C min^−1^ with a temperature range from ambient to 500 °C. Argon (Ar) was used as an inert environment. High-purity nitrogen was used as purging gas with a flowing rate for balance (40 mLmin^−1^) and a flowing rate for sample (60 mLmin^−1^). The pigment samples to be analyzed were loaded onto a platinum sample pan. The heating rate was dynamically and continuously modified in response to the sample’s mass loss rate changes, called controlled rate thermal analysis (CRTA).

The vibrating sample magnetometer (VSM) used was a VSM Model PAR155 (Lakeshore, Los Angeles, CA, USA). The samples were placed in a small Perspex holder attached to the end of a nylon rod. This was aligned in the magnetic field. Once the sample was positioned correctly, the magnetic field was increased incrementally until 12.5 kOe. This field strength was decreased and then increased back up again to 12.5 kOe. This gave a complete hysteresis loop to the samples.

### 2.4. Color Value Evaluation Method

**Figure 2:**
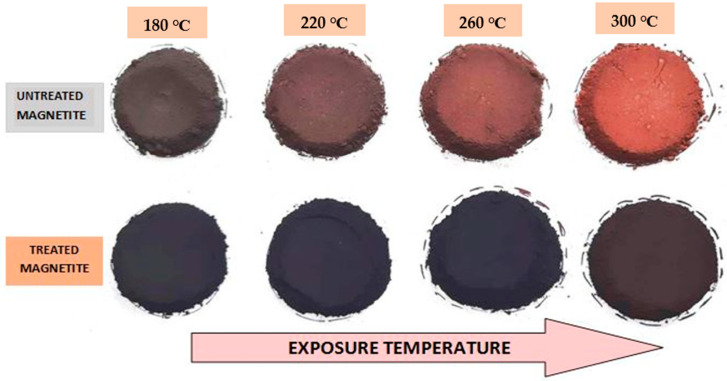
Photograph of untreated and SHMP-treated Fe_3_O_4_ powders at different temperatures for 30 min of in situ heat exposure, exhibiting visual color changes.

The treated IG Fe_3_O_4_ wet cake was collected from different beakers in Petri dishes and placed in a hot air oven at 50 °C for 24 h for drying. After complete drying, the samples were withdrawn from the hot air oven and were left to cool in the desiccator for 10 min. Figure 2 shows the calcined samples of bare IG Fe_3_O_4_ and treated IG Fe_3_O_4_ powders. The color change from black to reddish brown upon calcination is evident. The black color is retained very prominently in the case of the treated samples, even after calcination at 300 °C. Whereas, for the Fe_3_O_4_ bare samples_,_ the color changed from light brown at 180 °C to reddish-brown at 300 °C.

**Figure 3:**
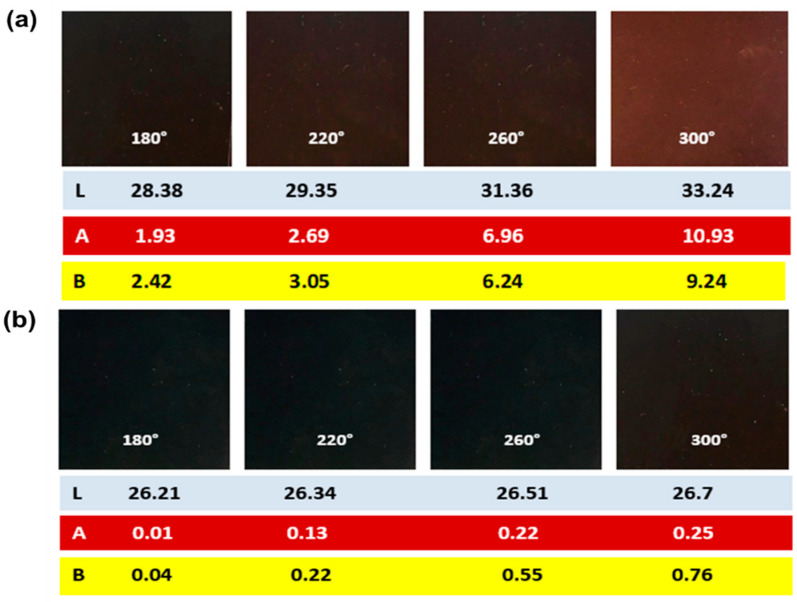
Photograph of painted panels depicting colorimetric measurements (L, A, B) of calcined: (**a**) bare Fe_3_O_4_; and (**b**) SHMP-treated Fe_3_O_4_ with 30 min of in situ heat exposure in an alkyd coating system.

The following test procedure was performed to measure the dE value in a paint system. In accordance with ISO 787-25 (2019) test standards, 11.5 g of pigment (IG Fe_3_O_4_) was taken in a cylindrical steel box, and 150 g of steel balls were added. Next, 10 g of alkyd resin (long oil alkyd) was added, along with 1 g of dispersant. The prepared mix was then placed for homogenous mixing for 30 min in an electric vibro shaker machine, also known as “vibroshaker”, from Irsha Engineering. Later, another 46 g of alkyd resin (long oil soya alkyd) was added with 1 g of the drying agent and further mixed in the vibroshaker for 5 min. With this, we formulated a black paint in the laboratory. The paint mix was applied on non-absorbent cardboard (or drawdown card) using a sheen applicator of 100 µm and was kept for drying at room temperature to achieve the desired dry film thickness. The drawdown cards were left overnight to dry, and after 24 h, the L, A, and B values were recorded using a color spectrophotometer (Premier Colorscan SS 5100A). Figure 3 shows the pictorial representation of absolute L, A, and B values of black painted panels applied over drawdown cards. The formula for calculating dE, the total color difference value, is defined in Equation (1):

(1) $$\mathrm{dE}=\sqrt{{\left(L_{2}-L_{1}\right)}^{2}+{\left(A_{2}-A_{1}\right)}^{2}+{\left(B_{2}-B_{1}\right)}^{2}}$$

Subscript 1 denotes the coordinate values for IG Fe_3_O_4_ at room temperature, the standard sample for comparison. Subscript 2 denotes the coordinate values of the calcined SHMP-treated Fe_3_O_4_ samples. If dE < 1, the color change cannot be perceived visually [38]. Here, we calculate the dE value by using CIE L*a*b coordinates. Colorimetric measurements in the CIELAB Cartesian system indicates lightness as L* (+L is lighter and −L is darker). Hue and saturation are quantified by chromatic a* (ranges from −a*, for green, to +a*, for red) and chromatic b* (ranges from −b*, for blue, to +b*, for yellow) [38].

An amount of 14 g of both bare and treated samples of IG Fe_3_O_4_ were kept in the hot air oven at various in situ heat exposure times (5, 10, 15, 20, 25, and 30 min). The experiments were conducted by varying the dosage of SHMP as IG Fe_3_O_4_: SHMP in the ratio of 85:15, 88:12, and 90:10, respectively, at a fixed exposure time of 5 min and comparing the dE value at varying temperatures (180, 220, 260, and 300 °C). The dosage of SHMP was fixed at 88:12 ratio of IG Fe_3_O_4_ and SHMP, respectively, based on the minimum DE value obtained, and subsequently, the exposure time and temperature were varied. Both the 88:12 and 85:15 ratios of IG Fe_3_O_4_ and SHMP performed well. The 88:12 ratio was selected for further study to obtain a cost-effective treatment.

Under oxidative conditions at temperatures above 180 °C, black iron oxide gradually changes its color from black to brown and then red, which is consistent with the phase change from magnetite (Fe_3_O_4_) to maghemite (γ-Fe_2_O_3_), and later hematite (αFe_2_O_3_) (>400 °C) [39]. For the surface-treated Fe_3_O_4_, this color change seems to be less. This color change could be further understood from colorimetric measurements (L, A, and B). A photograph depicting the colorimetric (L, A, and B) values of the calcined bare Fe_3_O_4_ and SHMP-treated Fe_3_O_4_ in an alkyd coating system is displayed in Figure 3. Upon calcination, the colorimetric parameters drastically increased for bare Fe_3_O_4_ samples. In contrast, the change was insignificant in the case of the treated Fe_3_O_4_ samples.

## 3. Results and Discussions

The chemical analysis of bare Fe_3_O_4_ and treated Fe_3_O_4_ were performed according to IS 44:1991 and IS 33:1992 test methods. The test results for the bare and SHMP-treated Fe_3_O_4_ confirms the international organization’s requirements for standardizing iron oxide pigments for paint specification, grade 1 black iron oxide Fe_3_O_4_, as presented in Table 1.

### 3.1. Scanning Electron Microscope and Energy Dispersive X-ray Spectroscopy Analysis of Bare IG-Fe_3_O_4_ and SHMP-Treated Fe_3_O_4_

**Figure 4:**
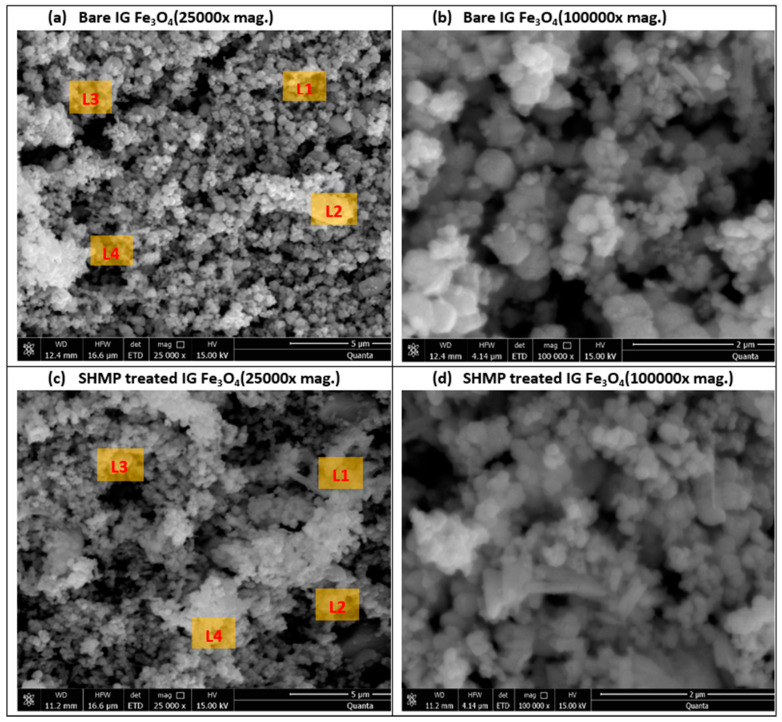
SEM microstructure images of (**a**,**b**) bare Fe_3_O_4_, and (**c**,**d**) SHMP-treated Fe_3_O_4_ samples at different magnification (25,000X and 100,000X).

The scanning electron microscope (SEM) images represented in Figure 4a–d, depicts the cubic inverse morphology of the bare and treated IG Fe_3_O_4_, which corroborates the presence of magnetite samples considered in this investigation. The EDX analysis (Table 2) reveals an increase in oxygen percentage and a presence of phosphorus (P), which portrays the SHMP treatment of the IG Fe_3_O_4_.

### 3.2. X-ray Diffraction Analysis of Bare IG-Fe_3_O_4_ and SHMP-Treated Fe_3_O_4_

**Figure 5:**
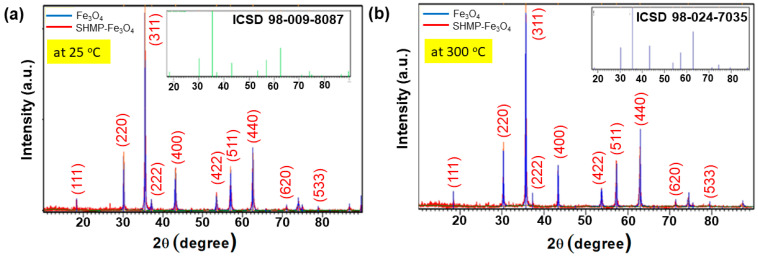
XRD patterns of bare Fe_3_O_4_ and SHMP-treated Fe_3_O_4_ particles at (**a**) room temperature, 25 °C (inset: XRD pattern of ICSD 98-009-8087) and (**b**) 300 °C calcined (inset: XRD pattern of ICSD 98-024-7035), respectively.

The XRD patterns for bare Fe_3_O_4_ and SHMP-treated Fe_3_O_4_ were recorded at room temperature, as depicted in Figure 5a. The peaks for both samples were identical with the standard peaks of Fe_3_O_4_ or magnetite from the ICSD database (Fe_3_O_4_ ICSD 158743), with a strong (311) peak at approximately 35.5°, accompanied by the (111), (220), (400), (511), and (440) planes of the cubic cell at 18.35°, 30.19°, 43.22°, 53.62°, 57.16°, and 62.77°, respectively. These planes could be indexed to face-centered cubic inverse spinel Fe_3_O_4_ with a lattice constant “a” to be 8.366 Å, as was also reported by Surowiec et al. (2017) [40].

Figure 5b illustrates that the peaks of calcined bare Fe_3_O_4_ at 300 °C shift upwards relative to the bare room temperature sample of Fe_3_O_4_, resulting in the change of lattice constant from 8.366 to 8.334 Å. A slight decrement of lattice constant was noticed due to the larger ionic radii of the Fe^2+^ cations in the crystal lattice of the Fe_2_O_3_.FeO crystal structure. In particular, the change in peaks or the diffraction patterns belong to γ-Fe_2_O_3_ or the maghemite phase (γ-Fe_2_O_3_ ICSD 247035) [41]. Simultaneously, calcined samples of SHMP-treated Fe_3_O_4_ at 300 °C show a similar face-centered cubic structure of the Fe_3_O_4_ lattice, which confirms that they acquired phase stability with this treatment. The SHMP particles potentially interact through the (111) plane of Fe_3_O_4_. We validated this based on reports [42] that assert that the (111) phase was mainly exposed to the environment and was favorable for binding to oxygen atoms. Moreover, we noticed that the (111) reflection of the calcined bare Fe_3_O_4_ particle demonstrates a decrease of the Fe_3_O_4_ share to 16%. Such a reduction in intensity was not observed for calcined SHMP-treated Fe_3_O_4_ samples.

Thus, it is evident from the XRD data that the calcined bare Fe_3_O_4_ undergoes a phase transformation from magnetite (Fe_3_O_4_) to maghemite (γ-Fe_2_O_3_), whereas the magnetite phase was retained in the case of the SHMP-treated Fe_3_O_4_ samples. Therefore, with these results, we can interpret that sodium hexametaphosphate retards the oxidation of iron and has a strong affinity to bind with the magnetite surface, which acts as a passivating layer for inhibiting the growth of γ-Fe_2_O_3_ (maghemite). The above-reported role of phosphate was also evident in the studies of Mohua et al. (2020) [43], Jerina et al. [44] and Lesia et al. (2000) [45].

### 3.3. Raman Spectroscopy Analysis of Bare IG-Fe_3_O_4_ and SHMP-Treated Fe_3_O_4_

**Figure 6:**
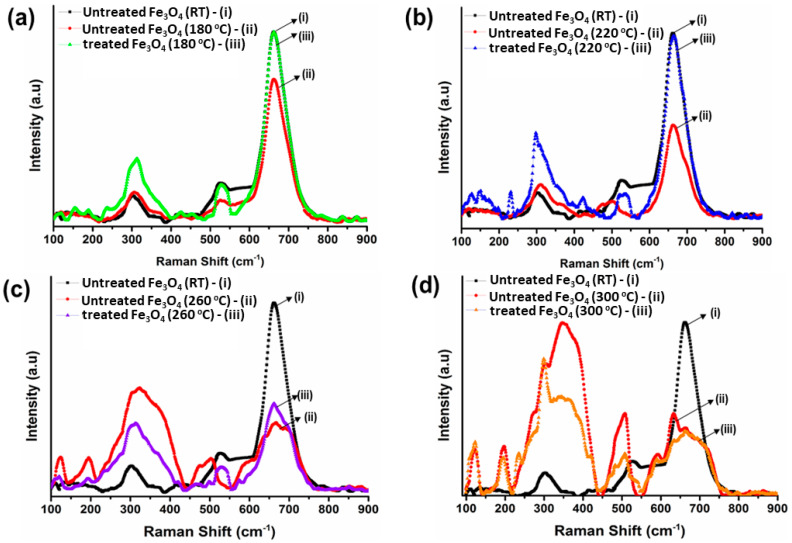
Raman spectra of bare and SHMP-treated Fe_3_O_4_ samples calcined at (**a**) 180 °C, (**b**) 220 °C, (**c**) 260 °C, and (**d**) 300 °C compared with bare Fe_3_O_4_ samples at room temperature, 25 °C.

The Raman spectrum for the room temperature sample of IG Fe_3_O_4_ is shown in Figure 6(ai)–6(di); whereas the Raman spectrum for the calcined samples of bare IG Fe_3_O_4_ (at 180, 220, 260, and 300 °C) and the calcined SHMP-treated Fe_3_O_4_ (at 180, 220, 260, and 300 °C) have been recorded in Figure 6(aii)–(dii) and Figure 6(aiii)–(diii), respectively. In Figure 6(ai)–(di), the bare IG Fe_3_O_4_ samples at room temperature yield vibrational modes at a laser power of 0.1 mW, observed as 312 cm^−1^, 540 cm^−1^, and 668 cm^−1^. The vibrational modes confirm the characteristics of the Raman vibration of magnetite [3,46,47]. Thus, Figure 6(ai)–(di) are considered as reference Fe_3_O_4_ spectrums to compare the Raman shift of the calcined samples of bare Fe_3_O_4_ and calcined SHMP-treated Fe_3_O_4_ samples. The detailed Raman shifts for all samples are presented in Table 3.

Figure 6(aii)–(bii) exhibits the Raman peaks for the calcined bare Fe_3_O_4_ at 180 and 220 °C, respectively. We observed a decrease in vibrational modes to 673 cm^−1^, 518 cm^−1^, and 330 cm^−1^ compared to magnetite peaks at room temperature. Simultaneously, the broadening and deterioration of vibrational modes in the 400–600 cm^−1^ region, along with the development of a new peak at about 700 cm^−1^ (in Figure 6(cii) for calcined bare Fe_3_O_4_ at 260 °C) is attributed to the initiation of the oxidation of magnetite (Fe_3_O_4_) to maghemite (γ-Fe_2_O_3_). Whereas, for bare Fe_3_O_4_ calcined at 300 °C, the prominent vibrational modes (704 cm^−1^, 512 cm^−1^, 365 cm^−1^) [48] of maghemite were observed due to the complete phase transformation of magnetite (Fe_3_O_4_) to maghemite (γ-Fe_2_O_3_), as also reported in our XRD study.

Interestingly, the observed peaks for calcined SHMP-treated Fe_3_O_4_ (at 180, 220, 260, and 300 °C), reported in Figure 6(aiii)–(diii), appeared to be very close to the room temperature peaks of Fe_3_O_4_, also reported in Table 3, which confirms the phase stability of magnetite with the SHMP treatment. 

### 3.4. Surface Area and Particle Size Analysis of Bare IG-Fe_3_O_4_ and SHMP-Treated Fe_3_O_4_

**Figure 7:**
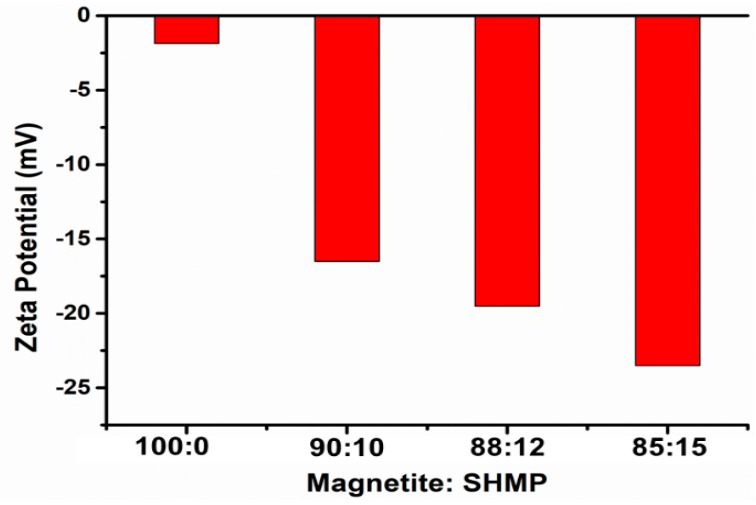
Zeta potential plot of bare Fe_3_O_4_ and SHMP-treated Fe_3_O_4_ by varying the Fe_3_O_4_ and SHMP ratio.

**Figure 8:**
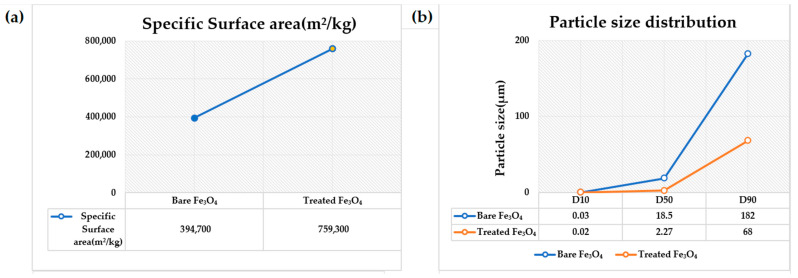
(**a**) Specific surface area, (**b**) Particle size distribution plot of bare Fe_3_O_4_ and SHMP-treated Fe_3_O_4_.

Figure 7 exhibits the zeta potential of bare and SHMP-treated Fe_3_O_4_ at different concentrations. The observed zeta potential was –1.86 for the bare Fe_3_O_4_ surface. With the SHMP treatment at different ratios (Fe_3_O_4_:SHMP: 90:10, 88:12, and 85:15), the Fe_3_O_4_ particle’s surface potential underwent a negative shift, which depicts a similar phenomenon to that reported in other literature [49], probably due to the adsorption of the negatively charged groups of SHMP components (H_2_PO^−4^ and HPO_2_^−4^) dissolved in the aqueous solution [50,51]. Thus, we could derive that the adsorption of long-chain polyphosphates on the Fe_3_O_4_ formed an outer-sphere complex [52]. This fact further validates that the induced stability, due to the steric repulsion of the negatively charged groups [53] present on the SHMP-treated Fe_3_O_4_ surface, contributed the de-agglomeration of the larger particles (essentially creating smaller particles that are readily dispersed). Thus, the D50 particle size (shown in Figure 8b) of around 18.5 µm for bare Fe_3_O_4,_ drastically reduced to 2.27 µm for the SHMP-treated Fe_3_O_4_ samples. We can confirm that SHMP restricts the aggregation of the Fe_3_O_4_ particles and reduces grain boundaries, maintaining homogeneity and well-dispersed particles.

We examined the particle size distribution for both bare and treated Fe_3_O_4_ pigments (Figure 8b). An increase in the specific surface area of 759,300 m^2^ kg^−1^ for the SHMP-treated magnetite particles, compared to 394,700 m^2^ kg^−1^ in the case of bare Fe_3_O_4_ particles, was observed, as shown in Figure 8a. The addition of SHMP avoided the agglomeration of the Fe_3_O_4_ particles, leading to a higher surface area, providing strength to enhance the thermal and magnetic properties of Fe_3_O_4_. The adsorption property of the polyphosphate component imparts a negative charge to the iron oxide particles, causing the de-agglomeration of the larger particles. The addition of SHMP reduced the surface tension at the solid–liquid interface, thereby increasing the nucleation rate, which resulted in finer particle size distribution, depicted in Figure 8b. The SHMP treatment made it possible to attain a uniform super-saturation at a lower surface tension, promoting a high nucleation rate and reduced particle size.

### 3.5. X-ray Photoelectron Spectroscopy Studies of Bare IG-Fe_3_O_4_ and SHMP-Treated Fe_3_O_4_

The results of the zeta potential measurements revealed that SHMP could adsorb on Fe_3_O_4_ surfaces. Therefore, to further understand the role of the interaction mechanism of SHMP with Fe_3_O_4_, XPS spectroscopy, a very surface-sensitive analytical technique, was conducted to measure the changes occurring on Fe_3_O_4_ surfaces with the addition of SHMP.

**Figure 9:**
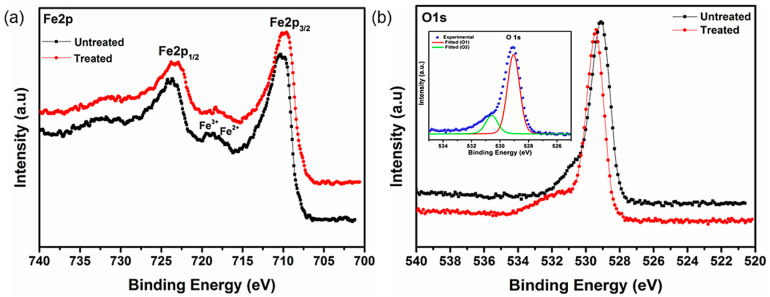
(**a**) Core-level spectra of Fe 2p on the Fe_3_O_4_ surface, and (**b**) core-level spectra of O1s Fe_3_O_4_ of both bare and SHMP-treated Fe_3_O_4_ samples (inset: deconvolution plot of O1s spectra).

The spectrum for the Fe 2p region, obtained from Fe_3_O_4_ particles, exhibits two peaks [54], 710.7 eV and 724.5 eV, which could be attributed to Fe2p_3/2_ and Fe2p_1/2_, while that of 710.22 eV and 723.37 eV are ascribed to Fe2p_3/2_ and Fe2p_1/2_ after treatment with SHMP, as depicted in Figure 9a. Figure 9b shows the O 1s spectra of the bare Fe_3_O_4_ and the SHMP-treated Fe_3_O_4_ consisting of a single peak at 529.1 eV and 529.5 eV, respectively.

The changes in the binding energy scales of the Fe 2p region exhibit a slight downward shift in energy from 710.7 eV to 710.22 eV. Interestingly, the O 1s region exhibits a slight upward shift in energy from 529.1 eV to 529.5 eV. Hence, the observed change in binding energy suggests orbital interaction resulting in successful SHMP layer incorporation on the Fe_3_O_4_ surface.

### 3.6. Thermal Analysis of Bare IG-Fe_3_O_4_ and SHMP-Treated Fe_3_O_4_

**Figure 10:**
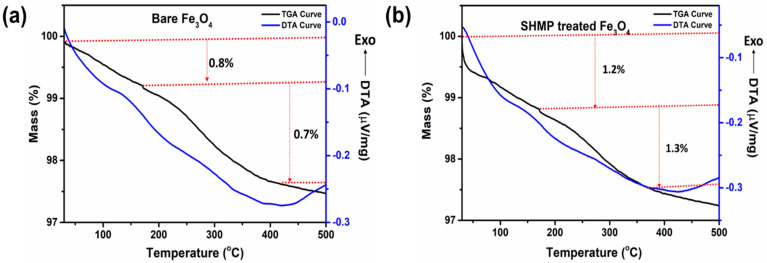
TG-DTA plots for (**a**) bare Fe_3_O_4_, and (**b**) SHMP-treated Fe_3_O_4_ samples, respectively.

The TGA curve of the bare Fe_3_O_4_ and the treated Fe_3_O_4_ samples measured in the range from 30 to 500 °C is shown in Figure 10. A minute mass loss (~1.5%) for the bare Fe_3_O_4_ sample may have originated from the surface moisture that was released once heated, as shown in Figure 10a, whereas the corresponding DTA curve exhibited a broad endothermic peak. Furthermore, TGA analysis of the SHMP-treated Fe_3_O_4_ sample indicated a loss of ~2.6% as recorded up to 500 °C (Figure 10b). The higher weight loss for this sample could possibly be due to volatile -OH groups in SHMP that may evacuate rapidly, and as a result, the nature of the TGA curve became stiffer than bare Fe_3_O_4_. An initial weight loss of ~1.2% with a broad endothermic peak at 110 °C could be associated with removing residual water and physically absorbing hydroxyl and SHMP from the surface of Fe_3_O_4_. The second step weight loss (~1.3%) was observed as being due to the decomposition of polyphosphate molecules on the surface. The decomposition process takes a long time due to the extended structure, resulting in broader DTA curves. Moreover, the polyphosphate molecules of SHMP were conjugated onto the surface of Fe_3_O_4_ particles through chemical bonding between oxygen atoms of polyphosphate and Fe_3_O_4_, which also reflects a broader DTA characteristic. For both samples, after 500 °C, insignificant weight loss was counted, and the curve started to flatten. Additionally, regarding the TGA characteristics, the treatment temperature of Fe_3_O_4_ was set at 180, 220, 260, and 300 °C.

### 3.7. Magnetic Property Analysis of Bare IG-Fe_3_O_4_ and SHMP-Treated Fe_3_O_4_

**Figure 11:**
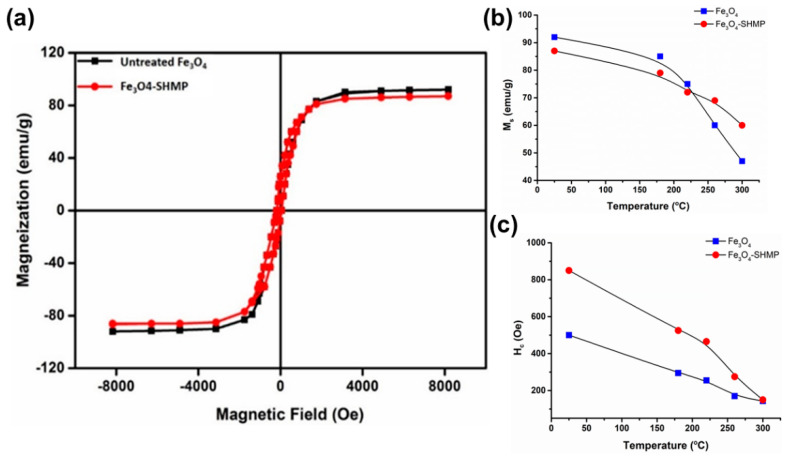
(**a**) Room temperature (25 °C) M-H (magnetization-hysteresis) loop of bare Fe_3_O_4_ and SHMP-treated Fe_3_O_4_, (**b**) plot of M_s_, and (**c**) H_c_ values for bare and SHMP-treated Fe_3_O_4_ at different temperatures.

Figure 11a represents the saturation magnetization (M_S_) of bare and SHMP-treated Fe_3_O_4_ measured at room temperature. The observed saturation magnetization values were 92 emu g^−1^ for the bare Fe_3_O_4_ and 87 emu g ^−1^ for the treated Fe_3_O_4_ samples. Both samples exhibited a weak hysteresis loop with ferromagnetic behaviour at room temperature. The coercivity value of the bare Fe_3_O_4_ (500 Oe) increased to 800 Oe for the treated samples. It is anticipated that the increase in coercivity may be due to the high magnetocrystalline anisotropy exhibited by the hexagonal microstructures caused by a coupling force, which was induced in the interface between the surface of the Fe_3_O_4_ and the SHMP molecule [55]. To understand the effect of calcination on their magnetization, both bare and treated Fe_3_O_4_ samples were measured, and their corresponding data are summarized in Table 4. In the case of calcined bare Fe_3_O_4_ at 180, 220, 260, and 300 °C, the Ms values were 85, 75, 60, and 52 emu g^−1^, respectively, as displayed in Figure 11b. Furthermore, a gradual drop in the M_S_ value was noticed with the increase in calcination temperature up to 300 °C, probably due to the formation of the maghemite phase. In the treated calcined samples, the coercivity (H_C_) value indicates that SHMP has imparted an extended phase stability on Fe_3_O_4,_ as shown in Figure 11c.

### 3.8. Paint Dispersion Property, and Colorimetric Study of Bare IG-Fe_3_O_4_ and SHMP-Treated Fe_3_O_4_

**Figure 12:**
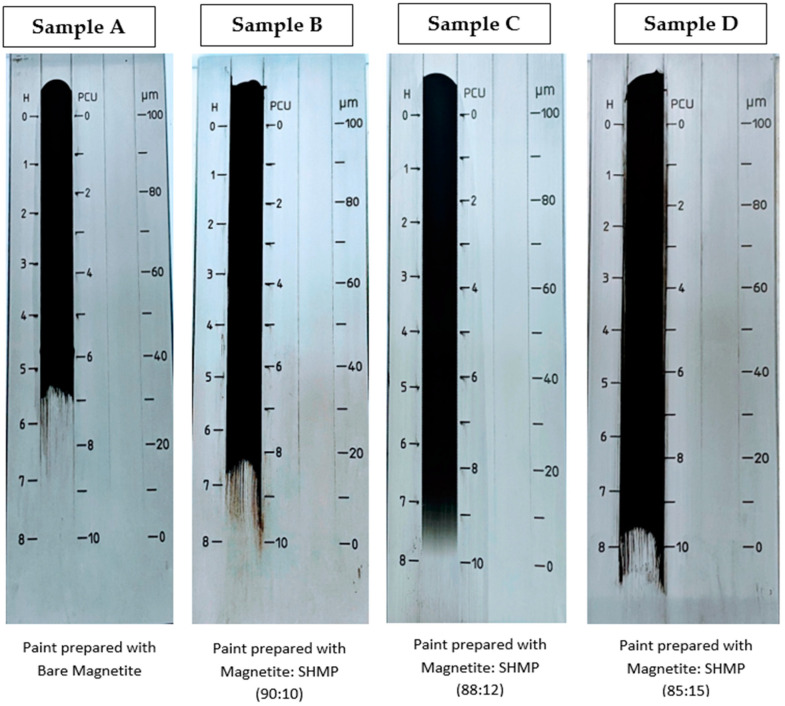
Photograph of the dispersion test of the paint system through the Hegman gauge for bare Fe_3_O_4_ and treated Fe_3_O_4_ at different concentrations.

Dispersion tests were conducted on the black paint prepared in the laboratory with the combination of bare Fe_3_O_4_ and SHMP-treated Fe_3_O_4_ at different concentrations denoted as Sample A, Sample B, Sample C, and Sample D. This paint dispersion test was examined with the help of a Hegman gauge instrument. On the left-hand side of the photograph, Figure 12 denotes the values in Hegman units (µm). The higher the Hegman unit value marked as H, the smaller the micron size of the paint particles, and the better the dispersion property of the paint would be. Figure 12 clearly shows that the dispersion is deficient in Sample A, wherein the black paint was prepared with bare Fe_3_O_4_ pigments with a Hegman unit value of 5.5. Sample C and Sample D gave the best results of 7.5+ Hegman units with the SHMP-treated samples of Fe_3_O_4_ at 88:12 and 85:15 ratios of Magnetite and SHMP, respectively. Interestingly, the micron particles of the black paint prepared with the SHMP-treated pigments reduced to 5 µm (Samples C and D) and 10 µm (Sample B), which could be due to the enhanced dispersion property of the pigment with the SHMP treatment. In contrast, it was above 30 microns µm for the black paint prepared with the bare pigment.

**Figure 13:**
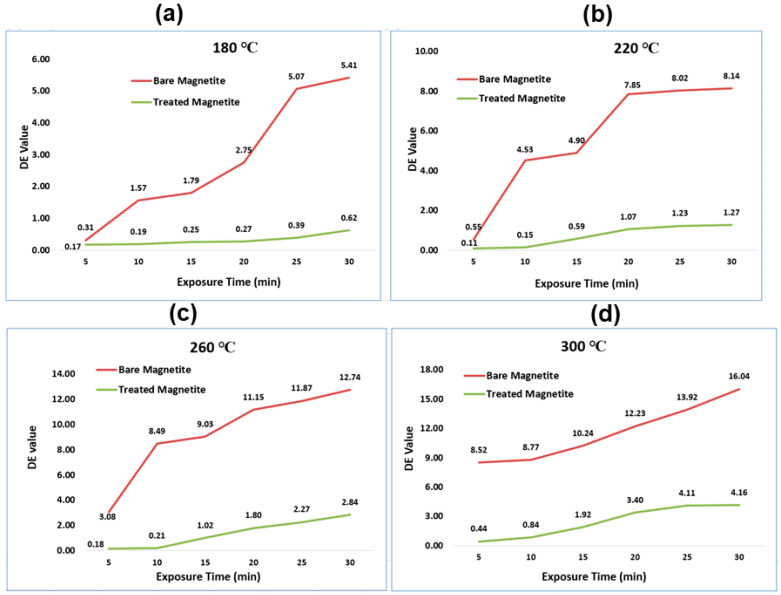
Plots depicting color difference delta-E (dE) values of bare and SHMP-treated Fe_3_O_4_ samples calcined at (**a**) 180 °C, (**b**) 220 **°**C, (**c**) 260 **°**C, and (**d**) 300 °C at different exposure times.

Figure 13a–d demonstrates the dE value of the black paint prepared with calcined bare Fe_3_O_4_ pigments compared with the black paint formulated with calcined SHMP-treated Fe_3_O_4_ pigments. The ratio of 88:12 (Magnetite: SHMP) was fixed based on the best paint dispersion property received and the maximum reduction in the dE values. The dE value of the black paint prepared with calcined bare Fe_3_O_4_ at 180 °C (Figure 13a), 220 °C (Figure 13b), 260 °C (Figure 13c), and 300 °C (Figure 13d) indicated a general increasing trend when compared with the room temperature samples. In the spinel FeO.Fe_2_O_3_ structure, under an oxidative condition, the FeO part was oxidized to form maghemite. This phase has a reddish-brown color. The dE value of the black paint prepared with SHMP-treated calcined Fe_3_O_4_ pigments was significantly low. It is evident from all the graphs in Figure 13 that the color deviation was dramatically reduced upon treatment. Thus, the surface treatment with SHMP retards black iron oxide’s brown coloration.

The color coordinates (L, A, and B) depicted in Table 5 demonstrate that the black paint prepared with the calcined bare Fe_3_O_4_ was less dark (high value of L*); whereas the black paint, formulated with the calcined SHMP-treated Fe_3_O_4_ appears to be darker (lower value L*). The chromatic parameters (a*, b*) of the calcined SHMP-treated Fe_3_O_4_ (as shown in Table 5) are consistent with the Fe_3_O_4_ pigments at room temperature, indicating limited surface oxidation with the treatment. In addition, the significantly higher chromatic parameters (a*, b*) of the calcined bare Fe_3_O_4_ at 260 and 300 °C are indicative of greater surface oxidation, which is consistent with the phase change from magnetite to maghemite.

## 4. Conclusions

In summary, efforts have been made to synthesize a robust thermal and color stable industrial-grade Fe_3_O_4_ black pigment by successfully employing the polymetaphosphate molecule-sodium hexametaphosphate (SHMP). By employing a series of physicochemical characterizations, we demonstrated the successful treatment of SHMP Fe_3_O_4_ pigments. Surprisingly, the calcined samples of the SHMP-treated Fe_3_O_4_ exhibited the distinguishable face-centered cubic structure of a Fe_3_O_4_ lattice, with no reduction in Fe_3_O_4_ share, as revealed by the X-ray diffraction study, which confirms that these samples acquired phase stability with this treatment. The higher negative zeta potential value and changes in the binding energy of the SHMP-treated Fe_3_O_4_ signifies that the surface adsorption of polyphosphate took place on Fe_3_O_4_. An increase in the coercivity value from 500 Oe (bare) to 800 Oe (treated) indicates an enhancement in the magnetic property of magnetite. The detailed dE analysis revealed SHMP’s role in retaining the color properties of the calcined Fe_3_O_4_ pigments. The results indicate that the surface treatment has been highly beneficial for the black oxide pigment to deliver stable colorimetric values and superior dispersion properties in the paint medium. This enhanced thermal stability property opens the way for a new approach to developing Fe_3_O_4_-based paint that retains its thermal properties, with cost-effectiveness for high-temperature paints and coating applications. The formulated SHMP-modified Fe_3_O_4_ pigment could be an eco-friendly alternative to the dyes and carbon black that contribute to paint contaminants and pollutants. We could further design a thermo-regulating paint that absorbs and releases heat inside brick buildings, keeping rooms warm whenever necessary by using excess energy.

## Figures and Tables

**Table 1:** Chemical characteristics of IG Fe_3_O_4_ and SHMP-treated Fe_3_O_4_.

Characteristic	Specification	Testing Method	IG Fe_3_O_4_	SHMP-Treated Fe_3_O_4_
Volatile matter % by mass	≤2.5	IS 33:1992	0.58	0.95
Residue on sieve % by mass on 45 µ sieve	≤0.5	IS 33:1992	0.35	0.33
Oil absorption	22–27	IS 33:1992	25.2	25.8
Matter soluble in water % by mass	≤1.0	IS 33:1992	0.28	0.26
Acidity (as H_2_SO_4_) or alkalinity (as NaOH) %age by mass	≤0.1	IS 33:1992	0.04	0.06
pH of aqueous extract	4–8	IS 33:1992	6.89	7.13
Total iron (as Fe_2_O_3_) % by mass	≥79%	IS 44:1991	79.2	79.1
Ferrous iron (as FeO) % by mass	≥20%	IS 44:1991	20	20.1
Carbonates (as CO_2_) % by mass	0.5	IS 33:1992	0.1	0.12

**Table 2:** EDX analysis as elemental weight (%) across different locations of bare and treated Fe_3_O_4_ sample.

Bare Fe_3_O_4_			SHMP-Treated Fe_3_O_4_		
Location (L)	O (%)	Fe (%)	O (%)	P (%)	Fe (%)
L1	20.47	77.89	32.19	1.01	65.68
L2	18.27	80.14	26.11	0.88	71.87
L3	15.60	82.78	22.31	0.90	75.63
L4	18.18	80.43	28.54	0.80	69.69
Mean	18.13	80.31	27.29	0.90	70.72

**Table 3:** Raman shift (cm^−1^) with assignment for bare IG-Fe_3_O_4_ and SHMP-treated Fe_3_O_4_.

Sample	Exposed Temperatures				
	25 °C	180 °C	220 °C	260 °C	300 °C
**Bare IG Fe_3_O_4_**	668 cm^−^^1^	673 cm^−1^	678 cm^−1^	697 cm^−1^	704 cm^−1^
	540 cm^−1^	518 cm^−1^	515 cm^−1^	513 cm^−1^	512 cm^−1^
	312 cm^−1^	330 cm^−1^	334 cm^−1^	341 cm^−1^	365 cm^−1^
**SHMP-treated Fe_3_O_4_**	668 cm^−1^	668 cm^−1^	670 cm^−1^	674 cm^−1^	674 cm^−1^
	540 cm^−1^	537 cm^−1^	540 cm^−1^	537 cm^−1^	520 cm^−1^
	312 cm^−1^	312 cm^−1^	315 cm^−1^	323 cm^−1^	312 cm^−1^

**Table 4:** Parameters obtained from VSM measurement for Fe_3_O_4_ and treated Fe_3_O_4_ samples at their ambient (25 °C) and different calcined temperatures.

Sample	Temperature (°C)	M_s_ (emu g ^−1^)	M_r_ (emu g^−1^)	H_c_ (Oe)
Fe_3_O_4_	25	92	34	500
	180	85	32	295
	220	75	30	255
	260	60	28	170
	300	47	25	143
SHMP-treated Fe_3_O_4_	25	87	32	850
	180	79	31.5	525
	220	72	31	465
	260	69	30	275
	300	60	29.5	150

**Table 5:** Colorimetric parameters of bare Fe_3_O_4_ and SHMP-treated Fe_3_O_4_ at different temperatures.

**Bare Fe_3_O_4_**					
**Color Coordinates**	**25 °C**	**180 °C**	**220 °C**	**260 °C**	**300 °C**
L*	26.9	28.38	29.35	31.36	33.24
a*	0.02	1.93	2.69	6.96	6.24
b*	0.26	2.42	3.05	6.24	9.24
**SHMP-Treated Fe_3_O_4_**					
L*	26.9	26.21	26.51	26.7	26.7
a*	0.02	0.01	0.13	0.22	0.25
b*	0.26	0.04	0.22	0.55	0.76

## Data Availability

The data presented in this study are available on request from the corresponding author.
